# A dataset of tumour-infiltrating lymphocytes in colorectal cancer patients using limited resources

**DOI:** 10.1093/database/baad089

**Published:** 2023-12-16

**Authors:** Anis Hasnaoui, Imen Helal, Zouhour Ben Azouz, Amira Hmidi, Raja Jouini, Aschraf Chadli-Debbiche

**Affiliations:** Faculty of Medicine of Tunis, Tunis El Manar University, Rue Djebal Lakhdar, Tunis 1006, Tunisia; Signals and Smart Systems Lab L3S, National Engineering School of Tunis, Tunis El Manar University, Campus Universitaire Farhat Hached B.P. n° 94 - ROMMANA, Tunis 1068, Tunisia; Faculty of Medicine of Tunis, Tunis El Manar University, Rue Djebal Lakhdar, Tunis 1006, Tunisia; Department of Pathology, Habib Thameur Hospital, Rue Ali Ben Ayed Montfleury, Tunis 1008, Tunisia; Signals and Smart Systems Lab L3S, National Engineering School of Tunis, Tunis El Manar University, Campus Universitaire Farhat Hached B.P. n° 94 - ROMMANA, Tunis 1068, Tunisia; Faculty of Medicine of Tunis, Tunis El Manar University, Rue Djebal Lakhdar, Tunis 1006, Tunisia; Department of Pathology, Habib Thameur Hospital, Rue Ali Ben Ayed Montfleury, Tunis 1008, Tunisia; Faculty of Medicine of Tunis, Tunis El Manar University, Rue Djebal Lakhdar, Tunis 1006, Tunisia; Department of Pathology, Habib Thameur Hospital, Rue Ali Ben Ayed Montfleury, Tunis 1008, Tunisia; Faculty of Medicine of Tunis, Tunis El Manar University, Rue Djebal Lakhdar, Tunis 1006, Tunisia; Department of Pathology, Habib Thameur Hospital, Rue Ali Ben Ayed Montfleury, Tunis 1008, Tunisia

## Abstract

In the realm of cancer research, specifically focusing on colorectal carcinomas (CRCs), a novel diagnostic test referred to as ‘Immunoscore’ (IS) has emerged. This test relies on assessing the density of tumour-infiltrating lymphocytes, specifically CD3 and CD8, in both the centre of the tumour (CT) and its invasive margin (IM). IS holds promise as a potential prognostic factor. A retrospective descriptive study was conducted within the Pathology Department of Habib Thameur Hospital in Tunis, Tunisia. The study’s aim was to evaluate the prognostic efficacy of IS for patients with CRC by means of a comprehensive survival analysis. This publication introduces the immunoscore in colorectal cancer (ISCRC) dataset, which was meticulously compiled during the aforementioned study. The ISCRC dataset comprises digital slide images sourced from biopsies of 104 patients diagnosed with CRC. Using the tissue microarray technique, an immunohistochemical investigation involving anti-CD3 and anti-CD8 markers was performed in regions designated as ‘Hot Spots’ within the CT and IM. The images were captured using a smartphone camera. Each marker’s percentage presence within its respective region was quantified. The IS was estimated utilizing a semi-quantitative method. The ISCRC dataset encompasses anonymized personal data, along with macroscopic and microscopic attributes. The captured images, acquired through manual efforts using smartphones, stand as a valuable asset for the advancement of predictive algorithms Importantly, the potential applications of these models extend beyond mere prediction capabilities. They lay the groundwork for innovative mobile applications that could potentially revolutionize the practices of pathologists, particularly in healthcare settings constrained by resources and the absence of specialized scanning equipment.

**Database URL:** https://figshare.com/s/5b4fa3e58c247a4851d4

## Introduction

Colorectal carcinoma (CRC) ranks as the third most prevalent cancer on a global scale, holding the unfortunate distinction of being the second leading cause of cancer-related deaths. In 2018 alone, a staggering 1.8 million new cases were reported, resulting in 861 663 fatalities (1). Within Tunisia, this cancer’s incidence continues to surge, posing a genuine and concerning public health challenge (2). The role of the pathologist transcends mere diagnosis, extending to the identification of prognostic indicators that are paramount for the therapeutic decision-making process related to CRC. While the tumour-nodes-metastasis (TNM) staging system has long stood as the gold standard for prognostic evaluation and clinical choices in CRC patients, its reliance on histopathological criteria, encompassing tumour invasion’s extent (T), lymph node involvement (N) and distant metastases (M), fails to fully capture the diverse clinical trajectories within patients sharing the same TNM stage (3, 4). This discrepancy has sparked a robust exploration of novel prognostic factors, with particular emphasis on the tumour microenvironment, notably tumour-infiltrating lymphocytes (TILs). Prior investigations have indicated that the infiltration of lymphocytes into the tumour environment holds predictive value for cancer progression, offering a potential prognostic determinant (5). TILs have been postulated to wield inhibitory effects on tumour growth, correlating with improved prognosis (6). In light of these insights, an immunological evaluation termed ‘Immunoscore’ (IS) has emerged. This assessment hinges on the immunohistochemical appraisal of total (CD3+) and cytotoxic (CD8+) lymphocyte densities in both the centre of the tumour (CT) and the invasive margin (IM) (7). Rigorous studies have illuminated the IS as an independent prognostic factor, linked to heightened survival rates and diminished recurrence risks (8). Validated on an international platform in 2018 through consensus (9), IS is believed to wield positive therapeutic implications, particularly for Stage II and III CRC cases. Its impact is anticipated to be integrated into forthcoming updates of the WHO classification.

Within Tunisia, the examination of IS remains conspicuously absent from anatomopathological reports. In this context, Helal *et al.* (10) embarked on a retrospective journey, evaluating the prognostic potential of IS across Stages I to IV CRC patients through a comprehensive survival analysis. During this study, an invaluable repository of metadata and histological images was meticulously curated. The focal objective of this publication is to present this dataset to the scientific community, thereby fostering the advancement of CRC research and computer-aided diagnostic endeavours.

## Value of the dataset

The dataset offers a comprehensive collection of images meticulously curated to facilitate the training of advanced models aimed at predicting the density of TILs within high-magnification field pathology slides sourced from patients diagnosed with CRC. These images, painstakingly captured through manual efforts employing smartphones, constitute a valuable resource for the development of predictive algorithms. The potential applications of these models extend beyond their mere predictive capacity. In essence, they lay the foundation for the creation of innovative mobile applications that could revolutionize the practice of pathologists, particularly in healthcare settings with constrained resources and the absence of specialized scanning equipment. By empowering pathologists with handheld diagnostic tools, this groundbreaking technology could bridge the gap between limited-resource environments and advanced medical diagnostics. The implications of such applications are nothing short of transformative. The technology, born from this dataset, would have the potential to redefine the standards of efficiency and time management in pathological assessments. It can significantly alleviate the burdensome demands of labour-intensive microscopic analysis, paving the way for accurate and swift evaluations that save precious effort and time.

## Data description

### Description of the dataset folders and files

The repository contains a zip folder packaged for download: ‘immunoscore.zip’. The zip folder holds one folder (images) and five files. As for the folder ‘images’, it contains 104 subfolders (one for each patient) named from 1 to 104. For each patient, four images are provided corresponding to two T-cell markers CD3 and CD8 for the CT and the IM. The naming rule of the pictures is: (ID)_(T-cell markers)_(area). For example, ‘1_CD3_CT’ corresponds to the image of Subject ID 1, stained with the CD3 marker and taken from the CT. When there are missing images, the corresponding patient subfolder is marked with an underscore.

A brief description of the dataset is shown in Table 1.

**Table 1.** Brief description of the dataset

No.	Particulars	Description
1	Number of patients	104
2	Pathology slides	TILs with immunohistochemistry staining
3	Total number of images	385
4	Image	Jpeg; 4608 × 3456 pixels; 72 dpi
5	Labels	Excel, csv
6	Dataset size	Size of images: 2.1–9.6 MB Images folder size: 1.94 GB Coding.txt size: 3.1 KB data_csv.csv size: 14.5 KB data_excel.xlsx: 39.2 KB labels.csv: 5.14 KB Missing.csv: 5.14 KB Immunoscore.zip folder size: 1.93 GB

**Figure 1.**
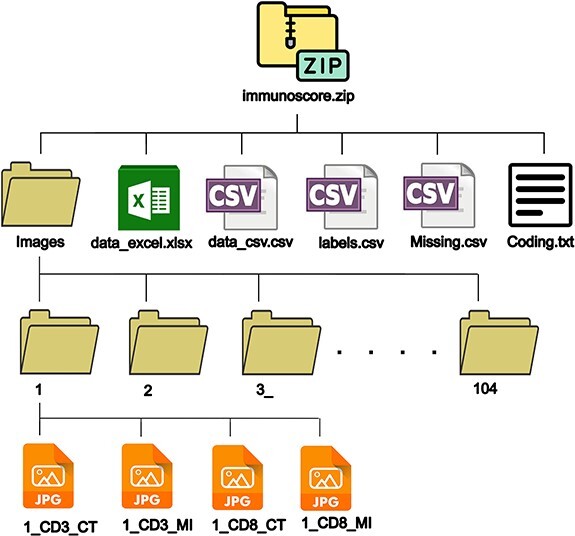
Dataset structure.

The dataset structure is shown in Figure 1.

Alt text: The structure of files and folders in the main zip file.

A brief description of the files contained in immunoscore.zip folder is shown in Table 2.

**Table 2.** Brief description of the files in immunoscore.zip

Name	File format	Description
data_excel	Excel	A file containing all collected data from the patient records, pathology reports and TIL scores. It comprises two sheets. The first sheet ‘data’ contains the actual data, and the second sheet named ‘coding’ contains the codes used to save the data.
data_csv	csv	A file containing the data from the first sheet of the excel file to provide more versatility to the user.
Labels	csv	A csv file containing the labels (density scores in %) of 385 images.
Missing	csv	A file containing missing images from patients’ subfolders (subfolders marked with underscores).
Coding	txt	A text file containing the coding from the second sheet of the excel file.

### Description of the images

In total, the dataset comprises a total of 385 images, meticulously organized within the 104 previously outlined subfolders. These images serve as a valuable resource for researchers aiming to train models capable of predicting the density of CD3+ and CD8+ TILs. Each image has been thoughtfully captured by pathology experts using a Huawei Nova 3i smartphone camera within the Department of Pathology at Habib Thameur Hospital in Tunis, Tunisia. The data collection spanned from 14 November 2020, at 9:55:11 A.M., to 19 June 2021, at 11:06:01 A.M. A description of the camera is shown in Table 3.

**Table 3.** Brief description of the camera

No.	Particulars	Description
1	Camera manufacturer	Huawei
2	Camera model	INE-LX1r
3	Software	INE-LX1r 9.1.0.265(C185E1R1P2)
4	Aperture value	f/2.2
5	Exposure time	1/50 s
6	International Organization for Standardization (ISO) sensitivity	ISO-160
7	Focal length	4 mm
8	Camera flash	None

All pictures were captured in the same location and using the same microscope. All images have a resolution of 72 dpi, with a width of 4608 pixels and a height of 3456 pixels, coupled with a bit depth of 24. The memory footprint of individual images spans a range from 2.1 MB to 9.6 MB.

**Figure 2.**
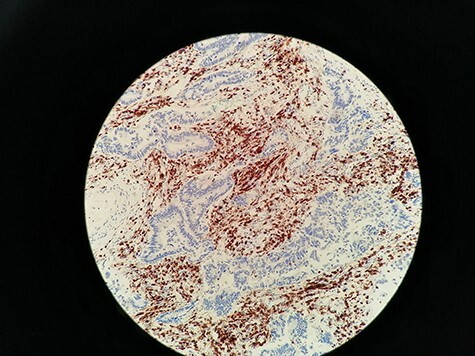
Immunohistochemistry staining for CD3+ TILs in 60_CD3_CT image. CD3 lymphocytes in CT are highlighted.

Images displayed immunohistochemistry staining for CD3 and CD8 at ×200 magnification. Target lymphocytes are marked with brown (positive) as shown in Figure 2.

Alt text: CD3 lymphocytes are marked with brown using immunohistochemistery.

## Materials and methods

### Patient cohort

This was a retrospective descriptive study of cases of CRC referred by the General Surgery Department of Habib Thameur Hospital and the Traumatology and Major Burns Centre. The cases were diagnosed at the Anatomical and Pathological Cytology Department of Habib Thameur Hospital and collected over a 3-year period (from 1 January 2014, to 31 December 2016).

We included cases of primary CRC, regardless of stage (I to IV), diagnosed at the Pathology Department of Habib Thameur Hospital during the study period and who underwent surgical resection along with lymph node dissection.

We did not include in the study:

- Cases of colorectal tumours with histological types other than carcinoma and
- Cases of CRC diagnosed via biopsies but the final resection specimen was not adressed to the department of pathology of Habib Thameur hospital. The decision to exclude biopsies from our study was based on the fact that biopsy samples were often unsatisfactory in terms of size and amount of tumour tissue.

We excluded from the study:

- Cases of middle and lower rectum carcinomas, where the tumour was located beyond 5 cm from the rectosigmoid junction. These cases underwent neoadjuvant treatment, which could influence the *in situ* immune response.
- Cases for which the slides of tumour specimens were not found in the archives of the pathology department of Habib Thameur hospital and
- Cases for which paraffin tissue blocks were depleted and therefore not available for further immunohistochemical study.

### Collection of clinical, pathological and follow-up data

Data collection was conducted using medical records and pathological reports and then organized into a descriptive framework. The dataset contains the following information (included in data_excel.xlsx and data_csv.csv files):

#### Personal data

Age, gender and medical history.

#### Macroscopic characteristics

- Tumour location.
- Tumour size.
- Macroscopic appearance.
- Distance from the resection margin.
- Presence of associated macroscopic lesions and
- Number of lymph nodes retrieved after careful mesocolon fat dissection.

#### Microscopic characteristics

- Histological type following the 2019 5th edition WHO classification.
- Histological grade (high and low grade).
- Tumour status (T), nodal involvement (N) and metastasis (M) according to the 2017 American Joint Committee on Cancer (AJCC)/Union for International Cancer Control (UICC) TNM classification for CRC.
- Presence of vascular emboli.
- Presence of perineural invasion and
- Stage.

#### Patient outcomes

The occurrence of recurrence or death, along with the dates of these events, was recorded. The follow-up duration was defined as the period between the diagnosis date and the date of death or, if not available, the date of the last update. Patients with available phone numbers were contacted.

### IS evaluation

We followed the recommendations published by Galon *et al.* (7), under the auspices of the Society of Immunotherapy and Cancer. This method was validated by a task force in which 22 global experts participated.

#### Slide review and selection of regions of interest

For each case, slides stained with haematoxylin and eosin (HE) were re-examined by a senior pathologist at low magnification (×40) to identify two areas with the densest lymphocytic infiltration, commonly referred to as ‘Hot Spots’. In each case, the first hotspot was located at CT, while the second was identified at IM. These areas were demarcated on the slide using a black marker.

The CT was defined as the central region of the tumour containing the stroma and tumour cells. The IM corresponded to the leading edge of invasion. Lymphocytic infiltration beyond the IM, as well as lymphoid nodules in the submucosa, was not included in the evaluation.

**Figure 3.**
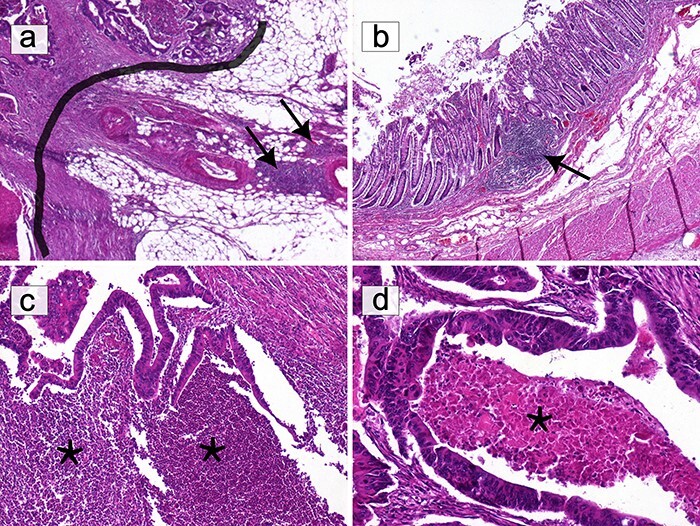
Selection of areas for T-cell quantification on HE slides. (**a**) Only T lymphocytes in direct contact with the IM (black border) were quantified. Lymphocytic infiltration beyond the IM, including lymphoid nodules (black arrows), was excluded (HE ×40). (**b**) Submucosal lymphoid follicles (black arrows) were not included in the count (HE ×40). (**c**) The infiltrate composed of granulocytes in abscessed areas (*) was not considered (HE ×100). (**d**) Areas of ‘necrosis’ (*) were not taken into consideration (HE ×200).

**Figure 4.**
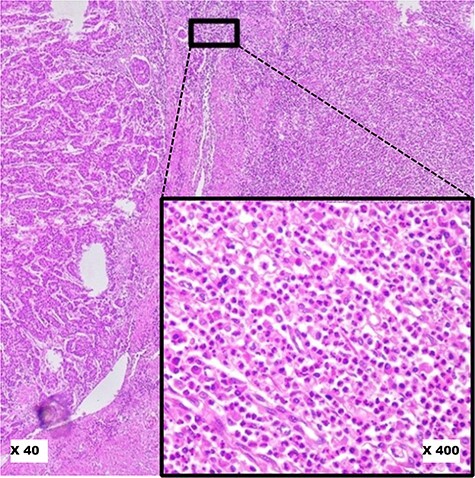
Pitfalls to avoid in selecting ‘Hot Spot’ areas on HE slides. This image shows a robust inflammatory response in the tumour microenvironment at low magnification (×40). At higher magnification (×400), it reveals a polymorphic inflammatory infiltrate particularly rich in neutrophilic granulocytes, which should not be considered.

Only lymphocytes were counted, excluding granulocytes, macrophages, mast cells and plasma cells. Necrotic and crushed areas were avoided during counting (Figures 3 and 4).

Alt text: HE slides highlighting areas to select or avoid for T-cell quantification.

Alt text: Polymorphic inflammatory infiltrate rich in neutrophilic granulocytes, to avoid when selecting hot spots.

#### Preparation of tissue microarrays (11, 12)

- Step 1: selection of donor blocks

After reviewing the slides, for each case, the blocks corresponding to the marked slides (‘Hot Spot’ zones) were retrieved from the archives. The tissue area to be sampled from each block was identified by aligning the paraffin block with the corresponding marked area on the slide of interest.

- Step 2: development of a recipient block map

Based on the number of cases included in the study, knowing that two samples were required for each case, the number of recipient blocks was predetermined. Each recipient block was organized according to a specific map. Ideally, the CT and IM areas of the same case were placed side by side, but due to technical considerations, a few samples from the same tumour were placed on different lines. The spot (1) was reserved for the external control, consisting of tonsillar tissue.

- Step 3: preparation of recipient blocks and construction of tissue microarrays (TMAs)

Similar to the TMA technique, two paraffin blocks per case were drilled, focusing on the ‘Hot Spot’ area. However, due to the absence of a microarrayer, paraffin blocks were perforated using a 5 mm-diameter biopsy punch. As per the predefined map, the tissue cores were re-embedded in each recipient block. The TMA blocks were then sectioned using a microtome at a thickness of 3–4 μm. Tissue sections were spread onto silanized slides to enhance tissue adherence and were placed in an oven at 37°C overnight. Morphological verification using HE staining was performed on one of the slides from each recipient block before the immunohistochemical study.

#### Study of CD3± and CD8± T lymphocytes by immunohistochemistry

An immunohistochemical study was conducted on TMA slides obtained from recipient blocks, enabling the analysis of multiple samples on a single slide. The study was performed using the avidin-streptavidin complex technique with the BOND-MAX automaton (Leica Biosystems). Anti-CD3 and anti-CD8 antibodies (Leica Biosystems) were used for the analysis of T lymphocytes. The evaluation of slides was carried out under an optical microscope by a senior pathologist.

#### Physical capture of images and determination of CD3± and CD8± TIL density

In most published studies, the determination of CD3+ and CD8+ T lymphocyte density is automated, following the 2018 international consensus (9). This consensus outlines a procedure where slides are first scanned at ×200 magnification, and then, using image analysis software, lymphocyte counting is performed within the ‘Hot Spot’ area, covering a 1 mm^2^ surface. In our case, and after consulting with Professor Pagès, the editor of the international consensus, we chose a semi-quantitative approach.

**Figure 5.**
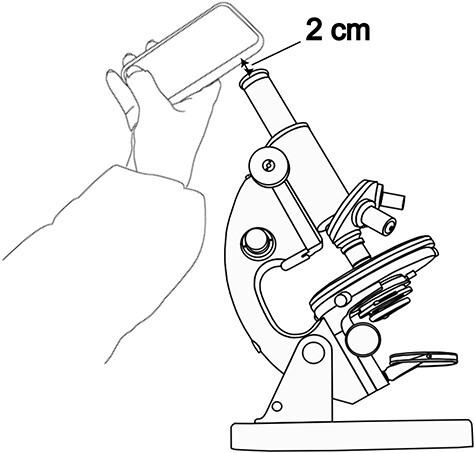
The photo-taking method.

**Figure 6.**
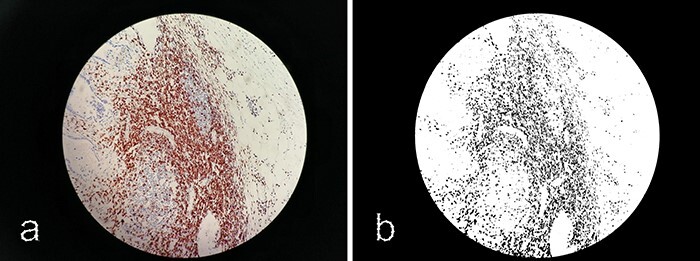
Processing images with ImageJ. (**a**) Original image 60_CD3_IM.jpg. (**b**) Processed image using ImageJ software.

The semi-quantitative approach steps are as follows:

- Measurement of a 0.95 mm^2^ area, corresponding to a field at ×200 magnification.
- For each case, in each region (CT and IM) and for each anti-CD3 and anti-CD8 staining, the areas where the lymphocytic infiltrate was maximal at ×200 magnification were identified.
- A photograph of each identified field was taken using a smartphone camera as shown in Figure 5. An optimal distance for capturing images was achieved by maintaining a consistent separation of 2 cm between the objective of the smartphone camera and the microscope’s ocular. This distance was supplemented with a tolerance of ±3 mm. No zooming was applied. This meticulous setup proved to be conducive to obtaining high-quality images.
- Utilizing the ImageJ freeware, images were transformed into an 8-bit format and subsequently refined through thresholding. The outcome of this processing is exemplified in Figure 6.
- Depending on the degree of lymphocytic infiltrate, a percentage was assigned to each image. The median of the infiltration percentages in each group (CD3_CT, CD8_CT, CD3_IM, CD8_IM) was calculated (Table 4) and used as a density estimation threshold: Density was considered ‘Low’ if it was below the median of the percentages and it was considered ‘High’ if it was higher than the median.

Alt text: The photo-taking method using a smartphone on top of the microscope’s ocular.

Alt text: Processing images with ImageJ software using thresholding to show only stained cells.

**Table 4.** Proportion of CD3+ and CD8+ lymphocyte densities along with their respective medians

	%CD3+		%CD8+	
Values	CT	IM	CT	IM
Minimum	2	1	0	0
Maximum	65	65	35	30
Median	20	17.5	10	9

#### Calculation of IS

We followed a method published by Galon *et al.* (7). For each marker (CD3 and CD8) and in each studied region (CT and IM), a score of 0 was assigned if the density was ‘Low’ and a score of 1 if the density was ‘High’. IS was then derived by summing these scores, resulting in a minimum score of 0 and a maximum score of 4:

I0: low densities of CD3+ and CD8+ TILs in both CT and IM.

I1: high density of one marker in only one region (CT or IM).

I2: corresponding to a score of 2.

I3: corresponding to a score of 3.

I4: high density of both markers in both CT and IM.

The IS was considered ‘High’ if it was equal to 3 or 4 and ‘Low’ if it was equal to 0, 1 or 2.

### Validation of images

In order to enhance the reliability of the label quality, a dual assessment approach was adopted. Each image was meticulously evaluated by both a resident and a senior pathologist. In cases where discrepancies emerged in the scores provided by these two professionals, a definitive score was established following a joint discussion to ensure consensus. The scoring procedure involved a comprehensive scrutiny of the images viewed under a microscope and their corresponding processed versions (utilizing ImageJ). Images afflicted by noise stemming from factors like blurriness or inadequate contrast were systematically excluded from the dataset to maintain data integrity. Folders with missing data are marked with an underscore.

## Limitations

**Figure 7.**
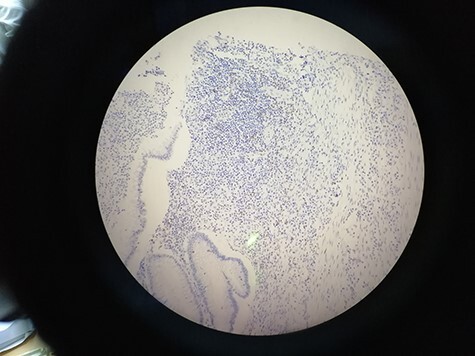
15_CD8_IM showing the deviation from the central alignment.

The images within our dataset were captured manually using a smartphone camera. It is important to note that a substantial portion of the images feature a significant background, and a few of them exhibit deviations from the central alignment of the microscope’s ocular, as illustrated in Figure 7. An additional factor that warrants consideration is the disparity in contrast and lighting observable among the images. This variance can be attributed to the diverse timeframes encompassing the acquisition of these images. Acknowledging these limitations is essential for a comprehensive understanding of the dataset’s scope and potential implications for the analysis. In forthcoming studies, our intention is to broaden the dataset’s horizons by incorporating a more extensive array of images encompassing varied conditions, such as lighting, and diverse materials including smartphones and microscopes. By embracing this expansion, we anticipate augmenting the dataset’s diversity and utility, rendering it an enriched and indispensable asset for researchers and pathologists alike.

Alt text: Deviation from the central alignment showing the background.

## Data Availability

The data underlying this article are available in the Figshare Repository, at https://doi.org/10.6084/m9.figshare.24007515.
